# Supplementary material for: Systematic identification of secondary bile acid production genes in global microbiome
Source: mSystems. 2024 Dec 17;10(1):e00817-24. doi: 10.1128/msystems.00817-24 (PMC11748489; doi:10.1128/msystems.00817-24)
Supplement: Supplemental Figures — Figures S1 to S4. [file msystems.00817-24-s0001.docx]

**Systematic identification of secondary bile acid production genes in global microbiome**

**Supplementary figures**

Table of contents

[**Supplementary Figure 1. Results of the screening for the secondary bile acid production gene catalog.** 2](#_Toc183963306)

[**Supplementary Figure 2. Phylogenetic trees based on the secondary bile acid genes distributed in different microbial kingdoms.** 3](#_Toc183963307)

[**Supplementary Figure 3. The weighted relative abundance of some major differential species.** 4](#_Toc183963308)

[**Supplementary Figure 4. The secondary bile acid production microorganisms composition in intestinal and liver diseases.** 5](#_Toc183963309)


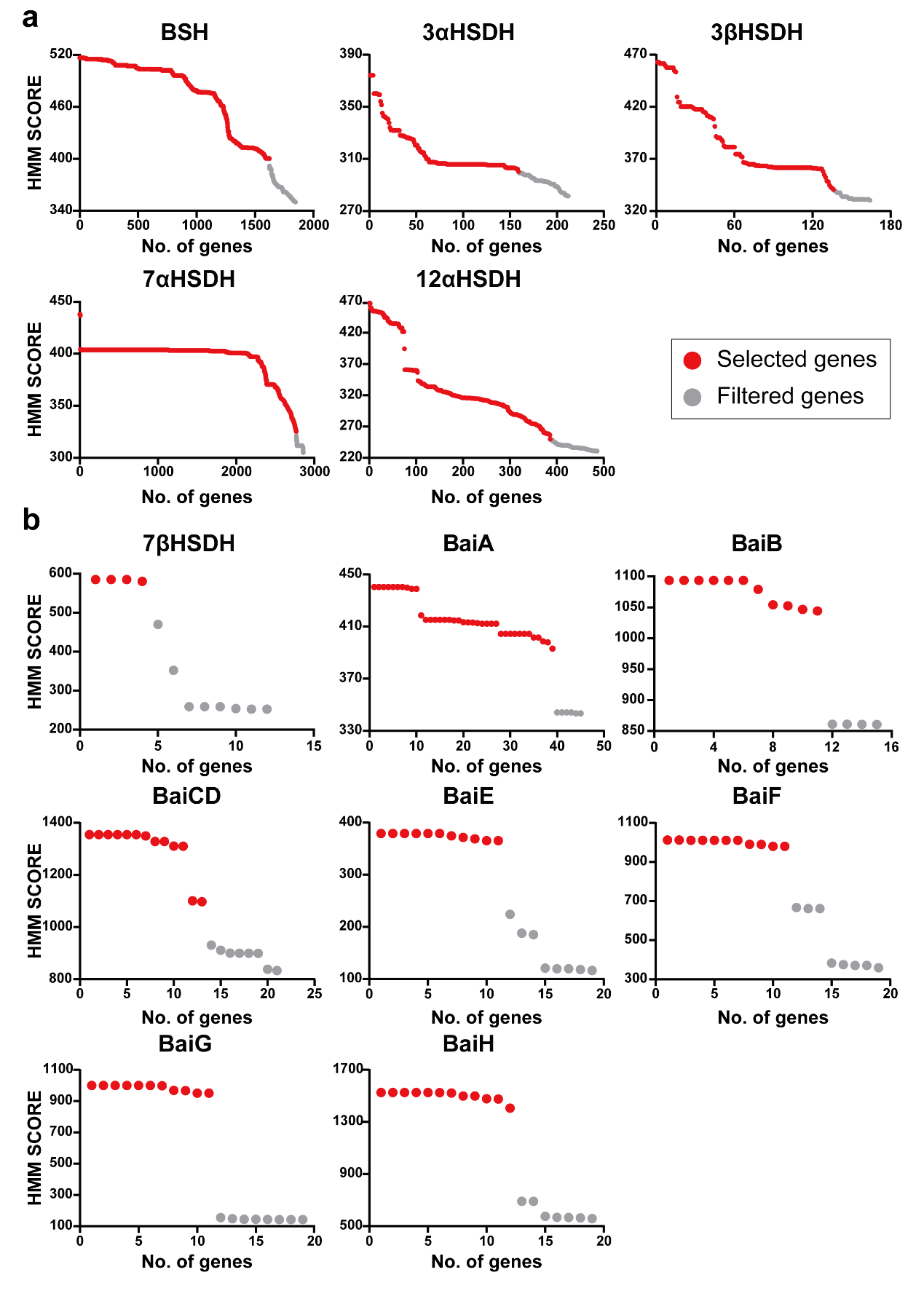


**Supplementary Figure 1. Results of the screening for the secondary bile acid production gene catalog.**

(**a**) (**b**) The candidates from hmmsearch are sorted according to their HMM score, and only genes in red are considered as the secondary bile acid production genes. For genes in (**b**), due to limited high-score results, genes from the PubSEED database were included as part of the secondary bile acid production gene catalog.


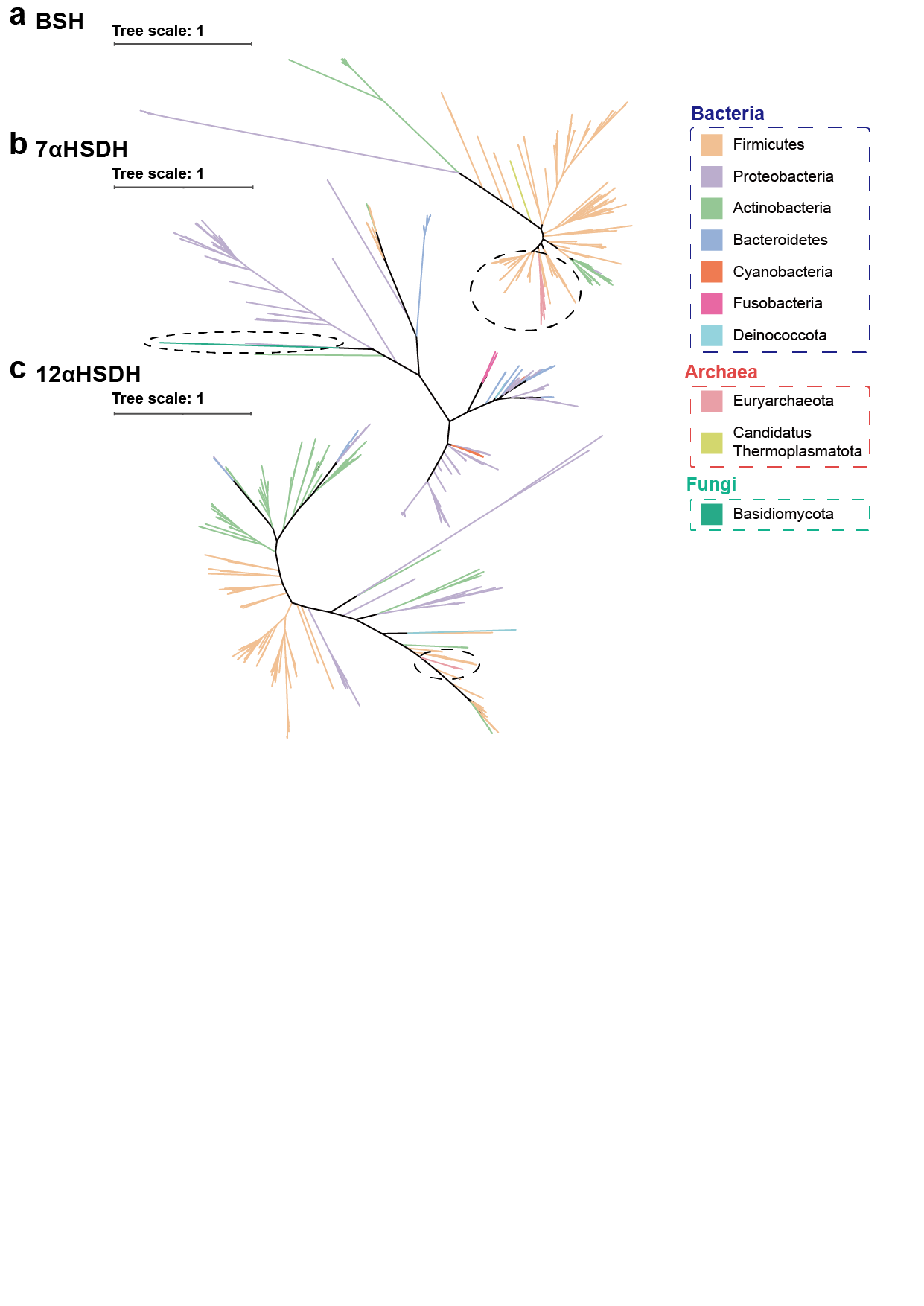


**Supplementary Figure 2. Phylogenetic trees based on the secondary bile acid genes distributed in different microbial kingdoms.**

Phylogenetic trees based on the non-redundant protein sequences of (**a**) bile salt hydrolase, (**b**) 7αHSDH and (**c**) 12αHSDH. The branch colors represent different phyla.


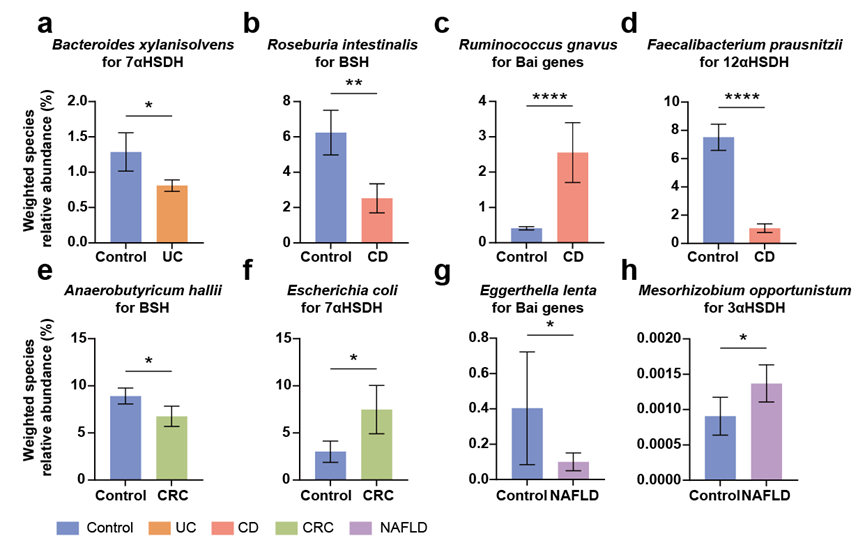


**Supplementary Figure 3. The weighted relative abundance of some major differential species.**

The bar plots show the weighted relative abundance of some major differential species. The bar colors represent different diseases. The definition of ‘Weighted species relative abundance’ is in equation (2). Data are shown as mean with standard error (SE). The statistical differences groups were determined by two-tailed Mann-Whitney U-test (UC, CD, adenoma, CRC) or paired t test (NAFLD), the p values were converted to asterisks (* for p ≤ 0.05; ** for p ≤ 0.01; *** for p ≤ 0.001 and **** for p ≤ 0.0001).


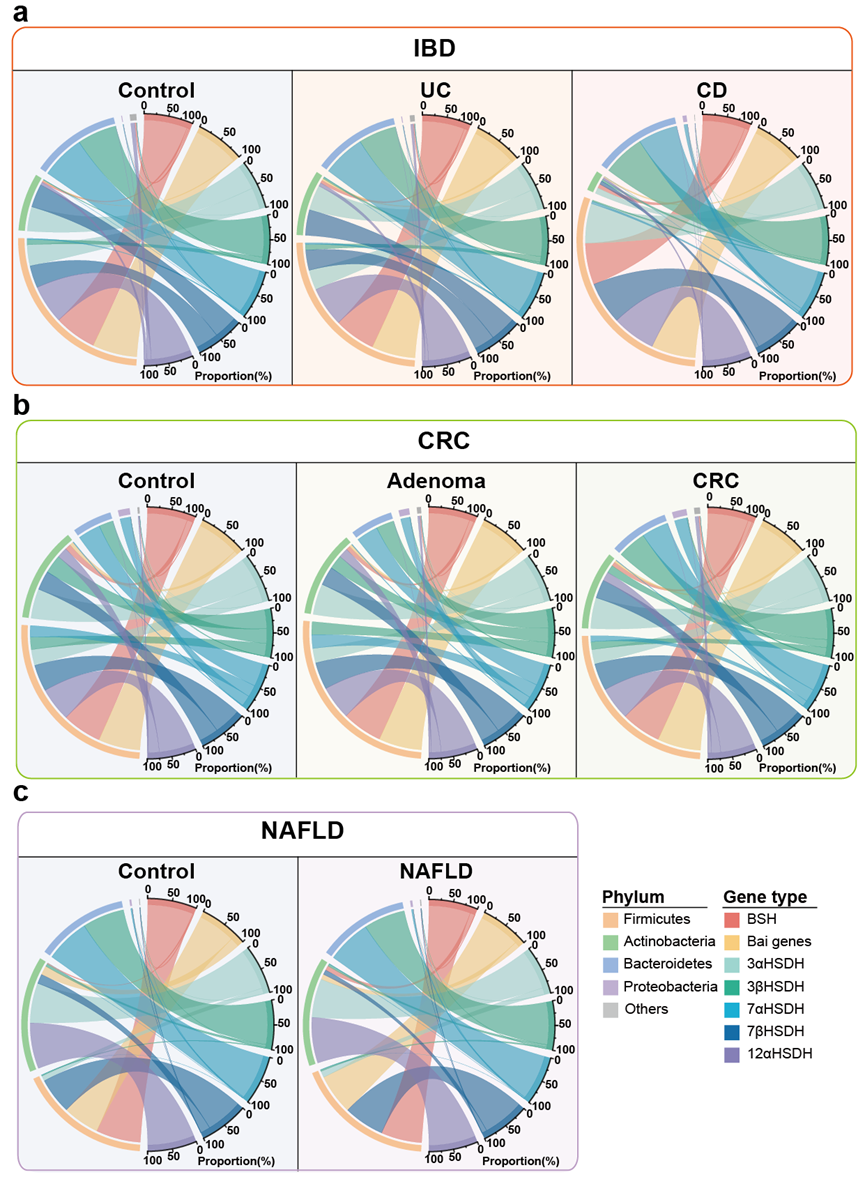


**Supplementary Figure 4. The secondary bile acid production microorganisms composition in intestinal and liver diseases.**

The chord diagrams show the weighted species abundance from different phyla related to secondary bile acid production. Maximum chord width corresponds to proportion of certain phylum and arrow color designates the gene type.
